# Modification of compensation mechanisms of outpatient services

**DOI:** 10.1186/1472-6963-14-S2-P16

**Published:** 2014-07-07

**Authors:** Marine Mardiyan, Armine Chopikyan, Aharon Barseghyan

**Affiliations:** 1 Healthcare Management Department, Yerevan State Medical University, Yerevan, Armenia

## 

Compensation in exchange for medical services is characterized as a unity of economic relations between medical institutions and funding side in exchange for the expenses of compensation during the provision of medical services. Compensation refers to compensation for expenses of the organization, not the individual departments and nor the medical worker [1-3].

In 1999 in the primary healthcare system there was a transition from the previous funding method toward the financing mechanism according to the number of served population (per capita), that is funding from the state budget of the RA was implemented compared with the average per capita amounts. Due to the implementation of this mechanism it became possible to realise cost containment, reduce administrative spending, increase efficiency in the use of financial resources. Despite the above mentioned advantages, financing mechanisms according to per capita are characterized by some drawbacks, particularly:

• absence of physician motivating mechanism,

• uncertainty of the number of people registered in the district, present and submitted patients number,

• absence of the system contributing to the increasing demand by population,

• groundless referrals to subspeciality consultations in order to increase their employment,

• groundless referrals to medical institutions,

• system inferiority of the payment of the immediate provider of medical service,

• unofficial payments etc.

Because of these deficiencies the funding mechanism does not provide people with the necessary primary medical aid and service with suitable volume and quality, as a result of which there was a decrease in population attendance in medical institutions; the number of preventative measures also decreased, the population had to apply for medical aid only in extreme cases, in addition to apply to its expensive component, that is to hospitals.

For that very reason, in order to make the medical care expenses more controllable and manageable it was necessary to choose such a compensating mechanism by the implementation of which it would be possible to realize the following actions:

• implementation of medical care and services volumes in the frame of state budget of the RA,

• cost containment and supervision increase in medical institutions,

• preliminary planning of medical aid volumes,

• more efficient and targeted use of provided financial resources.
